# The sit up test to exhaustion as a test for muscular endurance evaluation

**DOI:** 10.1186/s40064-015-1023-6

**Published:** 2015-07-02

**Authors:** Antonino Bianco, Corrado Lupo, Marianna Alesi, Serena Spina, Margherita Raccuglia, Ewan Thomas, Antonio Paoli, Antonio Palma

**Affiliations:** Sport and Exercise Sciences Research Unit, University of Palermo, Via Giovanni Pascoli, 6, 90144 Palermo, Italy; Department of Medical Sciences, Motor Science Research Center, School of Exercise and Sport Science (SUISM), University of Turin, Turin, Italy; Department of Psychology, University of Palermo, Palermo, Italy; Department of Biomedical Sciences, University of Padua, Padua, Italy

**Keywords:** Assessment, Strength, Inter-relation, Normative values

## Abstract

**Aims/Hypothesis:**

The aim of this study was to examine the sit up test to exhaustion as a field test for muscular endurance evaluation in a sample of sedentary people of both sexes.

**Methods:**

A cross-sectional study was performed. Three-hundred-eighty-one participants volunteered for the study (28.5 ± 10.0 years; 168.2 ± 8.9 cm; 65.1 ± 11.1 kg), of which 194 males (27.5 ± 10.2 years; 173.6 ± 7.0 cm; 71.2 ± 5.2 kg) and 187 females (29.6 ± 10.1 years; 162.6 ± 7.1 cm; 58.7 ± 8.9 kg). Each subject voluntarily and randomly performed: a sit up test (SUT), a push up test (PUT), and a free weight squat test (ST), all till exhaustion. A multiple regression analysis was adopted for data analysis. Subsequently a percentile model for muscle endurance was developed. The 25th, 50th, and 75th percentile were identified as upper limit for low muscular endurance, average muscular endurance, and lower limit for high muscular endurance, respectively.

**Results:**

Considering the sit up test as the dependent variable, the coefficients (R^2^ = 0.23; r = 0.49; p < 0.001), and (R^2^ = 0.31; r = 0.57; p < 0.001) emerged from a multiple regression analysis applied with respect to the push up test and the squat test, respectively. Gender stratification showed regression coefficients of (R^2^ = 0.19; r = 0.44; p < 0.001) for SUT vs. PUT, and (R^2^ = 0.30; r = 0.56; p < 0.001) for SUT vs. ST in male; and (R^2^ = 0.23; r = 0.49; p < 0.001) for SUT vs. PUT, and (R^2^ = 0.34; r = 0.59; p < 0.001) for SUT vs. ST in female.

**Conclusions/Interpretation:**

The SUT showed low inter-relation with the other proposed tests indicating that the adoption of a single test for the global evaluation of muscle endurance is not the optimal approach. Moreover, the SUT was found to be inexpensive, safe, and appropriate for core muscle endurance measurement for both male and female.

## Background

Many activities of daily living require a sustained effort exerted over a period of time. Therefore, muscle endurance is an important aspect of physical performance, and needs to be considered when assessing musculoskeletal functions (Ratamess 2012). Among these, core stability is progressively obtaining a fundamental importance in sport and in health promotion through fitness activities, being used as a daily muscle training session routine within the general athletic planning of most sports (Hibbs et al. 2008) and a majority of trunk conditioning routines within fitness centers (ACSM 2009). In particular, core stability is guaranteed by the contraction of all muscles located between knee and the sternum with a focus on the abdominal region (Hibbs et al. 2008), and refers to the ability of controlling the position and motion of trunk over pelvis to allow an optimal transfer of energy from the torso to body extremities during athletic activities (Kibler et al. 2006).

In line with the current scientific community debate on the opportunities of using field based tests (instead of laboratory ones) for the evaluation of motor skills (Lubans et al. 2011), field tests have been applied in order to monitor specific training. In particular, among these tests, weighted squat (ST) and push-up (PUT) tests find references promoting the evaluation of muscle performance leading to indirectly evaluate the corresponding muscle endurance by means of the relationship between number of repetitions and selected percentages of one repetition maximum (Shimano et al. 2006). Though, such tests performed to exhaustion as valuable evaluation of the muscle endurance for upper and lower body have been evaluated (Youdas et al. 2010). The body weight version to exhaustion of the ST test has been also adopted to directly evaluate the lower body muscular endurance (Willardson et al. 2008). At the same time, the PUT exercise, which is known to be one of the most effective and popular exercises for the strengthening of upper body muscles, is also traditionally performed as a standard measurement for the upper-body muscular endurance evaluation (Seo et al. 2013) by performing the maximum number of repetitions in 60 s (Cheema et al. 2013; Bedno et al. 2010). Though, it has recently also been considered to exhaustion to directly assess the latter parameter (Dwyer and Davis 2013). On the other hand, two protocols have been mainly adopted for the evaluation of core muscle endurance: the 30 and 60 s sit up test (SUT) (Blomqvist et al. 2013; Lucertini et al. 2013; Taeymans et al. 2009) which were adopted either in clinical, or sporting contexts (Mikkelsson et al. 2006; Frey and Chow 2006). Other outcomes show that the results of the 30-s SUT significantly vary according to the age of the participants, increasing or decreasing without any linear trend (Mikkelsson et al. 2006), whereas the 60 s SUT meanly reports 22 sit ups, limiting clear interpretations on the actual muscle strength endurance(Ingle et al. 2013). In addition, such short periods of time do not match with the definition of muscle endurance (Knudson and Johnston 1998) and it appears that longer “exposure times” are needed in order to properly evaluate abdominal muscular endurance.

As a consequence, the SUT executed to exhaustion represents the concept of an all-out test, and could be administered for a proper abdominal muscle endurance evaluation. Though this test has been poorly used, and no study has yet provided neither its validity or inter-relationship with other known muscle endurance tests.

Therefore, in consideration of the recognized validity of the ST (Dwyer and Davis 2013) and PUT (Youdas et al. 2010) to exhaustion as tests able to evaluate muscle endurance, the present study aimed to evaluate the relationships between these two practices and the SUT to exhaustion, to verify any relationships between different muscle compartments in the evaluation of muscle endurance and to examine if the SUT to exhaustion could be adopted as field test for muscular endurance evaluation. In addition, percentile discrimination will be performed in order to provide normative values, for all tests (i.e., 25th, 50th, 75th). For this purpose, it has been hypothesized that the SUT executed to exhaustion reports acceptable relationships (R^2^ > 0.30; r > 0.50; *p* < 0.001) with respect to both ST and PUT correspondent executions.

## Methods

### Study design

The STROBE statement for cross-sectional studies was adopted (Moreno-Ramirez et al. 2014; Vandenbroucke et al. 2014; Bolignano et al. 2013). The principles of the Italian data protection (196/2003) were guaranteed.

### Setting

In consideration of the recent scientific evidence which highlighted the effectiveness of the ST (Dwyer and Davis 2013) and PUT (Youdas et al. 2010) to exhaustion, in the present study, the latter executions were considered as measures of reference to establish an eventual SUT to exhaustion inter-relation for the evaluation of muscle endurance. Therefore, to achieve the aim of this study and to verify the experimental hypothesis, the individual outcomes of the SUT execution to exhaustion were compared to those of both ST and PUT.

Although these tests refer to different movements, and muscle group contractions, the PUT and ST trials performed till exhaustion were already considered for the muscle endurance evaluation in previous studies (Dwyer and Davis 2013; Chulvi-Medrano et al. 2012). For the same reason, it seems conceivable that SUT trails performed till exhaustion are linked to a reliable (and coherent to PUT and ST) muscle endurance evaluation. Furthermore, according to literature (Cohen 1992), the percentile discrimination could be applied to identify the specific grades of muscular endurance, and guarantee useful normative values for future studies. In particular, the ranges between the 1st and 25th (i.e., 25th), 25th and 75th (i.e., 50th), and 75th and 100th (i.e., 75th) percentile were able to classify the muscular endurance as low, average, and high level, respectively.

### Participants

All participants full filled a proper questionnaire containing two main sections: (1) information about the study design; (2) information about subjects’ health status and daily activities. Three-hundred-eighty-one participants volunteered for the study (28.5 ± 10.0 years; 168.2 ± 8.9 cm; 65.1 ± 11.1 kg). These where 194 male (27.5 ± 10.2 years; 173.6 ± 7.0 cm; 71.2 ± 5.2 kg), and 187 females (29.6 ± 10.1 years; 162.6 ± 7.1 cm; 58.7 ± 8.9 kg). The participants were recruited in the study according to the following inclusion criteria: (1) The obtaining of a medical examination that certified a good state of health; (2) were not regularly taking any medications (regular intake was defined as at least once a week); (3) were not during the first two days of the menstrual cycle; (4) were not under diet restriction regimen; (5) were beginners with sport and fitness activities.

### Variables

Each subject had to perform the following tests to exhaustion: SUT, PUT, and ST. Each test has been performed in a different day, within a period of 7 days. The random sequence was balanced as follow: each participant was randomly assigned to one of the three groups we created (Group A: ST, PUT, SUT; Group B: PUT, ST, SUT; Group C: SUT, ST, PUT). Two interval days were planned for each group A, B, C. The same operator administered each test, supervising the correct execution, and inviting participants to perform till exhaustion. In particular, the PUT could be considered correctly executed if the following criteria are guaranteed: The exercise was performed on a flat, stable surface, hands placed slightly wider than shoulder-width apart, and fingers pointed forward. Participants were instructed to maintain a neutral spine and feet together position throughout the entire movement. Once again, in order for the repetition to be recorded the correct depth needed to be met. Participants were instructed to lower the body until the chest was within 2 inch from the floor (Snarr and Esco 2013). The ST execution referrers to: place feet a little wider than shoulder-width apart. Extend the arms out straight. Initiate movement by inhaling and unlocking the hips, slightly bringing them back. Keep sending hips backward as the knees begin to flex. Bring hips parallel to ground. Return to standing position (Swinton et al. 2012). The correct SUT execution is provided by: The subject lay supine on the floor with 90° flexion in the knee joints, hands at the side of their head, and with elbows pointing straight forward. To do a correct sit-up execution the elbows should touch the knees and then go back so the shoulders touch the floor (Blomqvist et al. 2013). The number of repetitions performed was recorded to quantify the grade of endurance in each test. The condition of exhaustion (i.e., execution till failure) consisted to the inability to perform another repetition. In all tests, the participants were asked to constantly exercise through the concentric and eccentric phase of movements without any rest between the upper and lower grade of movements. The cadence of movements was controlled by a beep sounds coming from the Apps Runtastic Fitness© GmbH 2015.

### Statistical analysis

Firstly, the percentile distribution (i.e., 1st–25th, 25th; 25th–75th, 50th; 75th–100th, 75th) related to the number of repetitions performed during the SUT, PUT, and ST to exhaustion were calculated to provide the upper limit for low limit, average, and lower limit for high muscular endurance, respectively. Afterwards, to verify the grade of inter-relation of the three observed tests (i.e., SUT, PUT, ST), a linear regression analysis was adopted using the software STATISTICA v.8.0 (StatSoft©, Tulsa, USA). In particular, the SUT was considered as dependent variable. Statistical significance was set at an alpha level of *p* < 0.05. The Bland–Altman plots were obtained through the GraphPad Prism 5 for Windows (San Diego, CA, USA).

## Results

**Figure 1:**
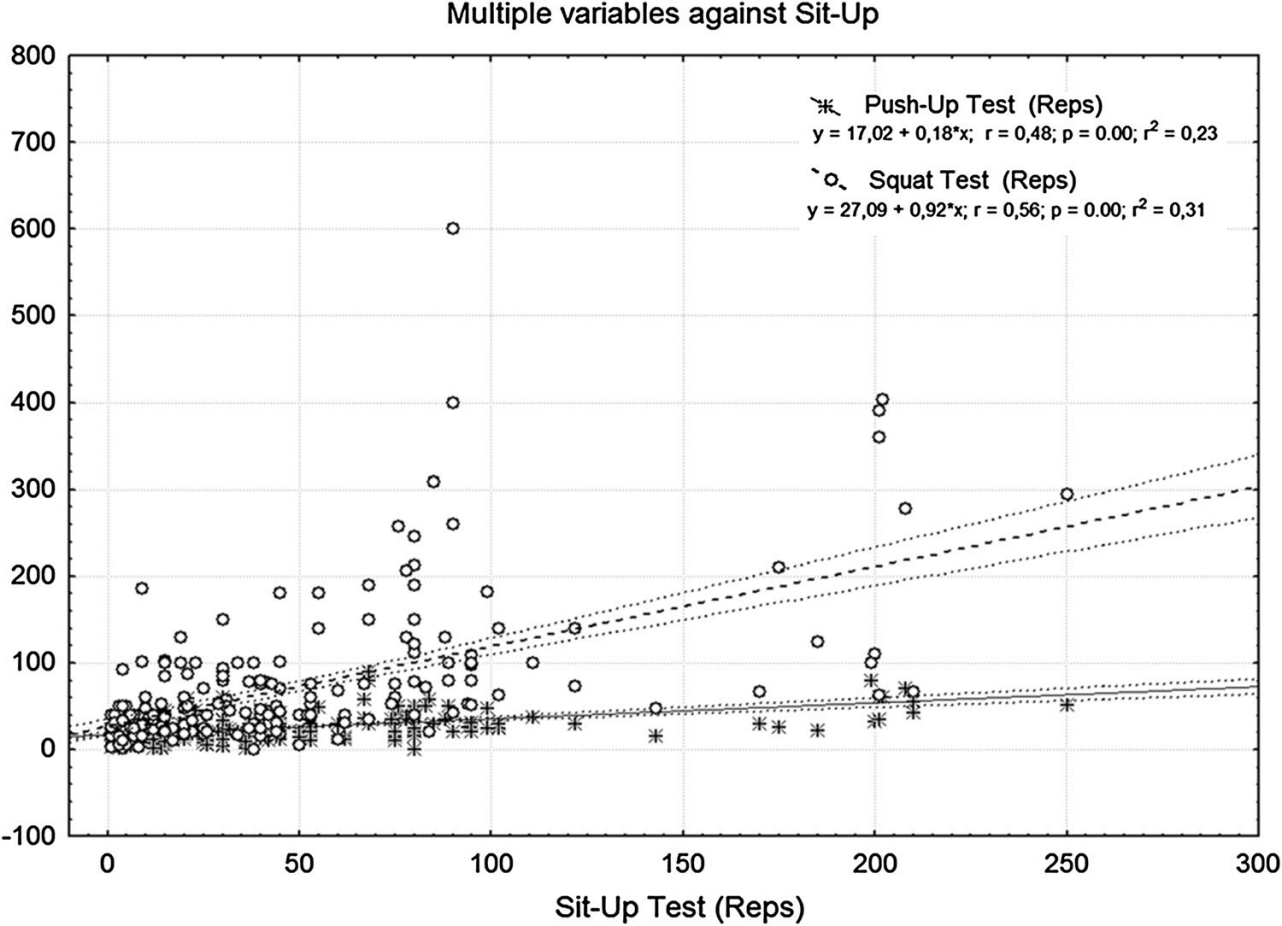
Linear regression analysis of the sample (*n* = 381).

All participants to the study meanly performed 44.73 ± 41.62, 25.28 ± 16.05, and 68.37 ± 68.91 repetitions during SUT, PUT, ST, respectively. Stratification of gender, male performed 53.29 ± 45.43, 32.62 ± 17.26, and 90.87 ± 84.20 repetitions in SUT, PUT, and ST, whereas, females performed 35.86 ± 35.26, 17.10 ± 10.13, 45.05 ± 34.10 repetitions emerged for the same three tests, respectively (Table 1). The regression coefficients of the entire sample are shown in Figure 1 and Table 2, whereas the gender stratification regression coefficients are reported in the Table 2.

**Table 1:** Number of repetitions performed

	All participants			Male			Female		
	SUT	ST	PUT	SUT	ST	PUT	SUT	ST	PUT
Reps	44.73 ± 41.62	68.37 ± 68.91	25.28 ± 16.05	53.29 ± 45.43	90.87 ± 84.20	32.62 ± 17.26	35.86 ± 35.26	45.05 ± 34.10	17.10 ± 10.13

**Table 2:** Multiple regression (i.e., sit up vs. push up; sit up vs. squat) values for all participants

	All participants			Male			Female		
	*p*	*r*	R^2^	*p*	*r*	R^2^	*p*	*r*	R^2^
Sit up vs. push up	<0.001	0.49	0.23	<0.001	0.44	0.19	<0.001	0.49	0.23
Sit up vs. squat	<0.001	0.57	0.31	<0.001	0.55	0.30	<0.001	0.59	0.34

All participants n = 381, male n = 194, and female n = 187.

**Figure 2:**
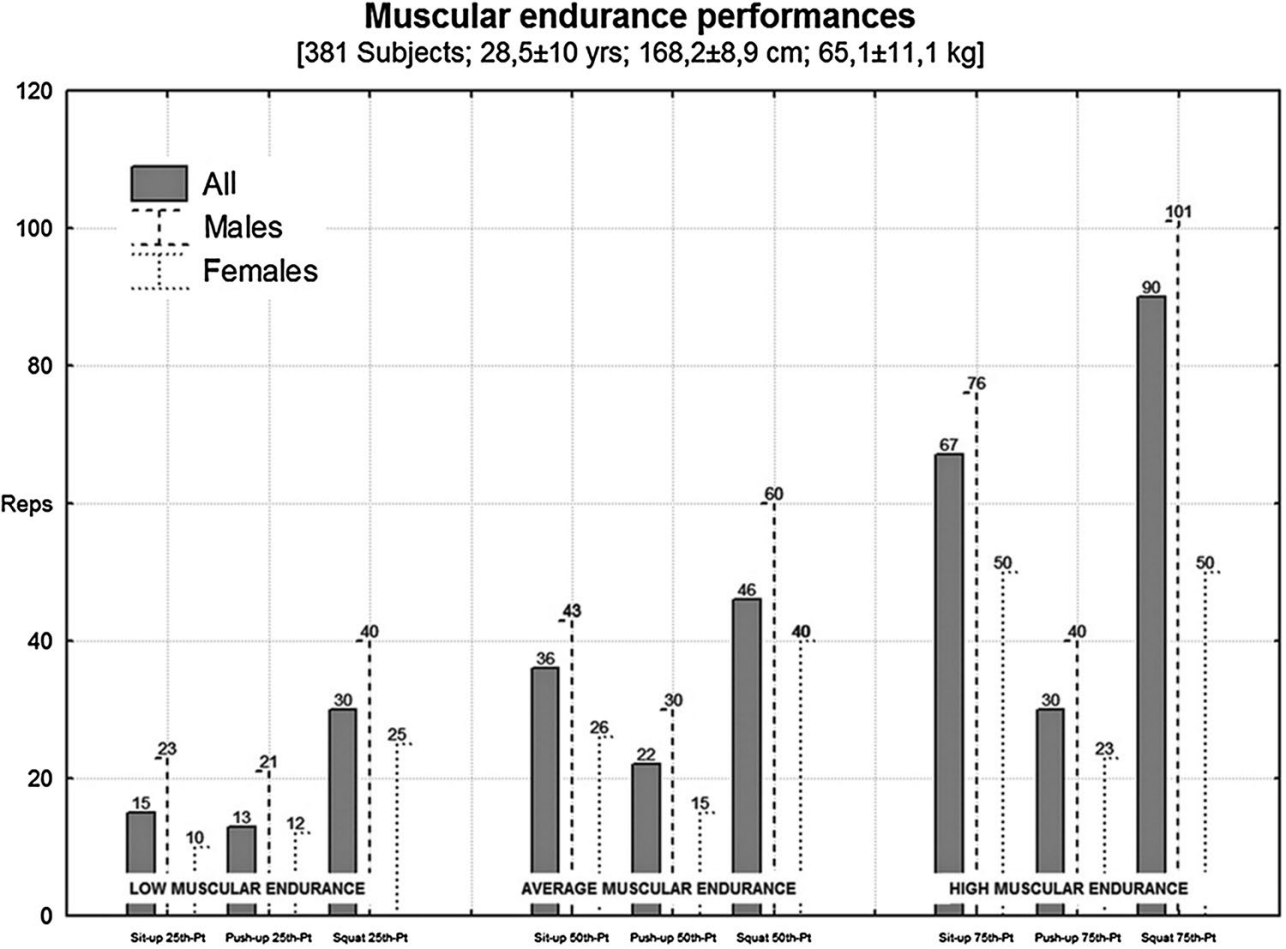
Percentile value model. At each test are assigned three categories, low muscular endurance, average muscular endurance and high muscular endurance. The 25th, 50th, and 75th percentile were identified as upper limit for low limit, average, and lower limit for high muscular endurance, respectively.

A percentile model for muscle endurance on the base of the present cohort was showed in Figure 2. In particular, the percentile discrimination was applied to identify the low, average, and high muscular endurance levels.

**Figure 3:**
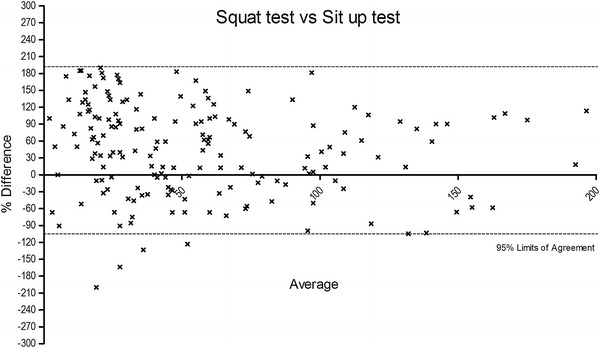
The figure shows the % of difference vs. average of squat test vs. sit up test. A number of seven data points are outside the axis limits.

**Figure 4:**
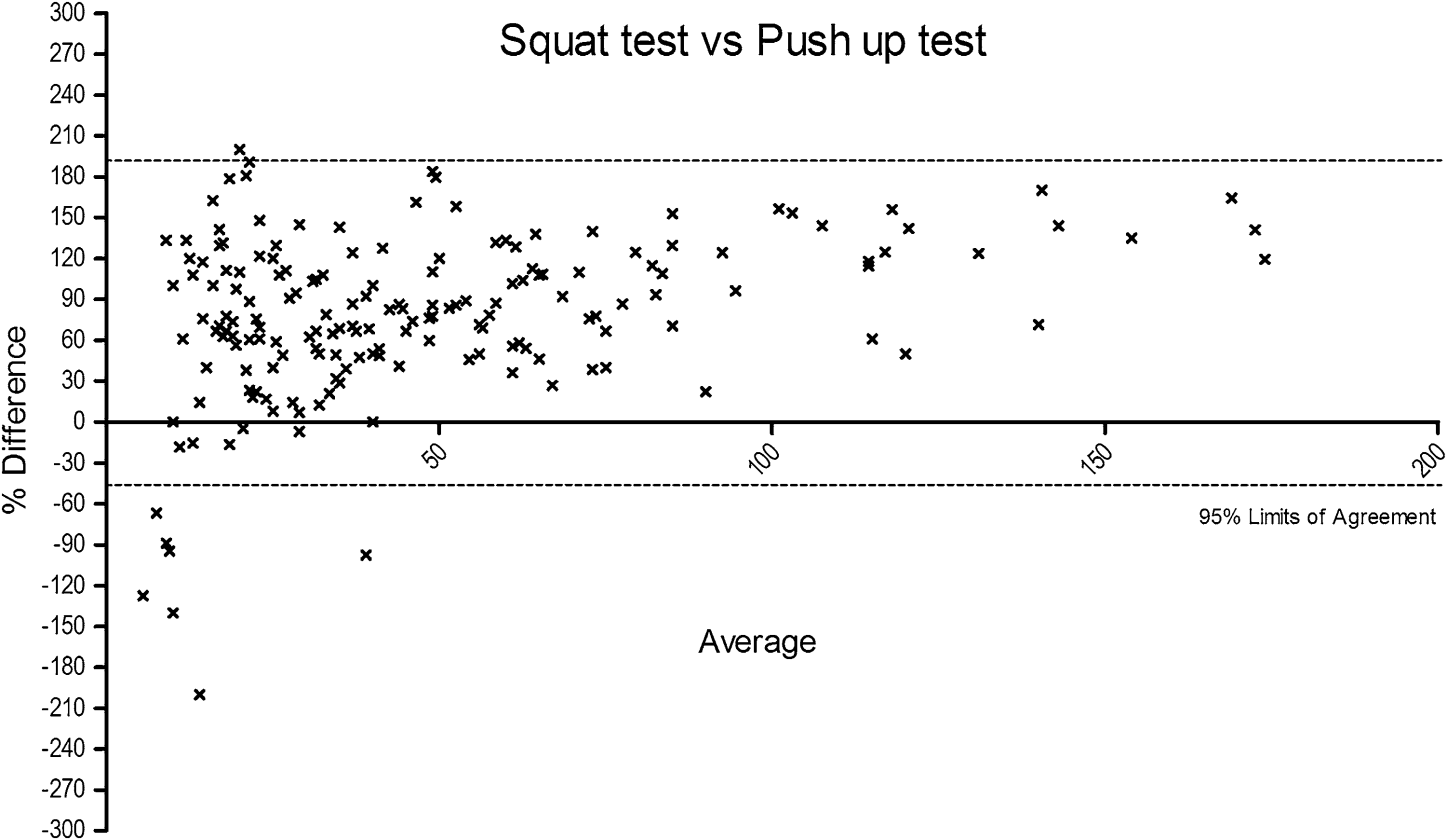
The figure shows the % of difference vs. average of squat test vs. push up test. A number of five data points are outside the axis limits.

**Figure 5:**
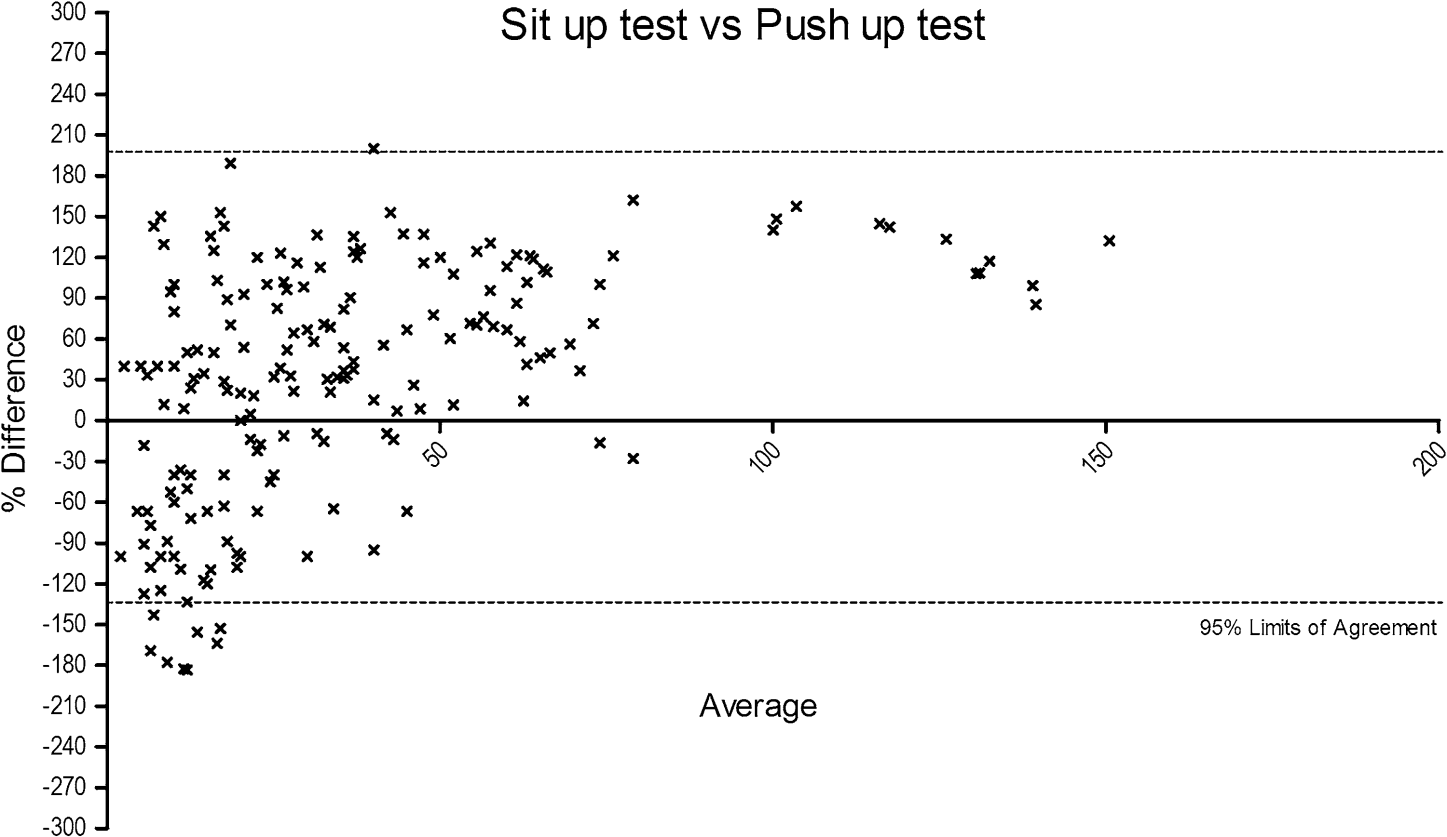
The figure shows the % of difference vs. average of sit up test vs. push up test.

The Bland–Altman plots were obtained in order to determine the bias between the tests and the limits of agreement (Figures 3, 4, 5). A the end of this section we may state that our experimental hypothesis could be partially accepted, because only the relationship between SUT and ST tests to exhaustion reported acceptable coefficients considering the entire participant sample (*p* < 0.001; r = 0.57; R^2^ = 0.31), as well as stratified for both genders (male: *p* < 0.001; r = 0.55; R^2^ = 0.31; female: *p* < 0.001; r = 0.57; R^2^ = 0.31). In regard to SUT vs. PUT, tests only approached (*p* < 0.001; r = 0.49; R^2^ = 0.23), reporting not fully appreciable values for both male (*p* < 0.001; r = 0.44; R^2^ = 0.19) and female (*p* < 0.001; r = 0.49; R^2^ = 0.23) participants.

## Discussion

Considering that the concept of muscle endurance can fall into the context of resistance training (Stratton et al. 2004) (i.e., exercise specifically designed to enhance muscular strength and endurance; Exercises demanding muscle work with over weights or specific body weight exercises), and is defined as the ability of a muscle or group of muscles to sustain repeated contractions against a resistance for an extended period of time (Brown 2013), all the tests to exhaustion considered for the present study meet these criteria, fully guaranteeing the substation of repeated contractions, which are requested to exactly evaluate muscle endurance. Some studies (Blomqvist et al. 2013; Lucertini et al. 2013; Taeymans et al. 2009) consider the 30 s SUT as a measure of muscular endurance, whereas others (Mikkelsson et al. 2006; Frey and Chow 2006) have adopted the 60 s SUT to evaluate such motor skill. However, according to the above definition of muscular endurance, if a subject is able to perform such task for a period of time greater than that proposed by the time subjected tests, the muscular endurance could not be properly evaluated, while a test to exhaustion could fulfill such definition exactly.

Although, tests to exhaustion have been poorly studied, functional measures for upper and lower body strength, using both PUT and ST, have been investigated (Butler et al. 2010; Freeman et al. 2006). In particular, it has been shown that the PUT is a validated measure for upper body muscular endurance (Youdas et al. 2010) and is an effective method for the activation of trunk and limb muscles (Howarth et al. 2008; Youdas et al. 2010), thus highlighting it, as an ideal field-based instrument, which is also easy to administer. In addition, for the lower body muscular endurance, the squat exercise has been recognized as a functional and safe maneuver that closely resembles the very common activity of moving (Munich et al. 1997), thus making it as an administrable and reliable test. Finally, also the sit up is coherently characterized by an execution which involves various muscle groups and is easy to administer (Escamilla et al. 2010). In regard to the main aim of the study, the inter-relation is partially satisfying underlining that the SUT could be a predictive measure for the ST but not for the PUT. Single measures would better suit the purpose of an accurate muscle endurance evaluation. All the considered tests in this study have common points like the involvement of large muscle groups during executions (Youdas et al. 2010; Munich et al. 1997), and the simplicity and usefulness of administration. Therefore, on the base of these considerations, the SUT to exhaustion could also be framed for muscle endurance evaluation, and considering the outcomes of the regression analysis showing sufficient correlations, SUT to exhaustion could be largely adopted in a field-based context, showing it as a test for muscle endurance assessment. Secondly, a percentile model based on the present data was created. This percentile model also briefly clarifies the grade of muscle endurance classifying the results in three main comprehensible categories: low, average and high muscle endurance. In terms of practical implications, these preliminary results partially confirm our hypothesis, defining muscle endurance not strictly inter-related. The SUT, though, was found to be inexpensive, safe, and a repeatable measure of core muscle endurance for both male and female. Such test could be assessed in different contexts from clinical conditions as a measure of core residual function as well as in sport environments for the evaluation of athletes involved in strength and endurance disciplines in youth and advanced athletes. Moreover, for muscle endurance, and in particular for the above proposed tests, few references have been discussed in the literature. Therefore, despite the above reported need of further research to consolidate procedures and data, the scales reported in the present study offer useful references for the muscle endurance related to the ST, PUT and SUT, thus allowing strength and conditioning trainers to easily classify the physical skills of each beginner fitness attendee according to the identification of three categories (i.e., low, average, high muscular endurance).

## Conclusions

The SUT showed relatively low inter-relation with the other proposed tests indicating that a single test for the global evaluation of muscle endurance is not appropriate. The physiological characteristics of each muscle district influences the outcomes of the tests. Although, the SUT being easy to administer, and of low costs could be effectively framed within the context of muscle endurance evaluation. Finally, the detection and setting up of reference values are constantly evolving, representing the main topics for a lot of textbooks, congresses, workshops, and round tables that are organized all over the world. For this reason, methods and results reported for this first approach on the evaluation of muscle endurance related to the ST, PUT and SUT should stimulate the promotion of further research on this topic, tending to determine a higher or lower stability of the proposed testing evaluations.
